# Effect of White Tea on Leptin and Asprosin Levels in Rats Feeding a High-Fat Diet

**DOI:** 10.3390/life14121548

**Published:** 2024-11-26

**Authors:** Adnan Yilmaz, Merve Nur Toraman, Sibel Mataraci Karakas, Zulkar Ozden, Esra Pinarbas, Tolga Mercantepe

**Affiliations:** 1 Department of Biochemistry, Faculty of Medicine, Recep Tayyip Erdogan University, 53020 Rize, Türkiye; m.nurtoraman@gmail.com (M.N.T.); sibel.karakas@erdogan.edu.tr (S.M.K.); esra.pinarbas@erdogan.edu.tr (E.P.); 2 Department of Histology and Embryology, Faculty of Medicine, Recep Tayyip Erdogan University, 53020 Rize, Türkiye; zulkar.ozden@saglik.gov.tr (Z.O.); tolga.mercantepe@erdogan.edu.tr (T.M.)

**Keywords:** asprosin, antioxidant, high-fat diet, leptin, obesity, white tea

## Abstract

Background: Currently, obesity affects over 600 million individuals and is responsible for numerous severe health conditions, particularly diabetes and metabolic syndrome. The objective of our study was to examine the impact of white tea, known for its potent antioxidant properties, on the reduction in body weight as well as the levels of leptin and asprosin. Methods: A total of 72 male Sprague–Dawley rats were randomly assigned to 9 groups, with each group consisting of 8 rats. The groups were partitioned into two in order to examine the preventative and therapeutic effects of white tea on obesity. During this study, the case groups were administered white tea together with a high-fat diet, whereas the positive control group was administered orlistat along with a high-fat diet through oral gavage. After the experiment concluded, the levels of leptin, asprosin, and insulin hormones were evaluated in serum samples collected from rats using the ELISA method. Results: The findings demonstrated that the administration of white tea led to a significant decrease in body weight, serum leptin, and asprosin levels, as well as oxidative stress indicators, in rats that were fed a high-fat diet. Conclusions: Utilizing natural chemicals, such as white tea, which possess minimal side effects and have powerful antioxidant activity, can mitigate the detrimental consequences associated with obesity.

## 1. Introduction

Obesity, characterized by the abnormal or excessive accumulation of body fat, currently affects over 600 million people globally and has been identified as a pandemic by the World Health Organization (WHO). It is well known that obesity leads to numerous serious health problems, such as cardiovascular disease, dyslipidemia, insulin resistance, diabetes, stroke, gallstones, fatty liver, obesity hypoventilation syndrome, sleep apnea, cancers in the long term, and shortened lifespan. Obesity and the treatment of related health problems constitute a global burden [1,2,3,4]. Obesity arises from a multifaceted interaction of genetics and environmental factors, including diet, exercise, psychology, and behavior [5]. Obesity typically arises from an imbalance where the amount of calories ingested exceeds the amount of calories burned [6]. Poor dietary habits, characterized by excessive consumption of foods rich in fat, protein, and calories and disturbances in the body’s circadian rhythm, are significant contributors to obesity. The modern lifestyle prevalent in society has facilitated the development of this problem [7,8].

Adipose tissue functions as an active endocrine organ that releases a range of hormones known as adipokines. These hormones have a role in regulating metabolism and inflammation, and they may serve as a connection between obesity and the risk of developing diseases [9]. Adipose tissue releases bioactive substances that communicate with other organs involved in metabolism, including muscle, liver, pancreas, and brain, through endocrine processes. This communication helps regulate overall metabolism. Extensive evidence indicates that the defective production, control, release, and communication of adipokines are linked to the onset of obesity and related conditions [10].

Food intake is a physiological process that seeks to sustain the body’s energy equilibrium. Several behaviors, signals, and physiological mechanisms regulate food choices and intake. Providing the energy requirement is a fundamental prerequisite for sustaining life. Humans, similar to numerous other species, fulfill their energy needs by consuming food. Hormones that influence appetite can either increase (oroxygenic) or decrease (anorexigenic) energy intake [11,12]. Leptin is a hormone that has a role in controlling energy balance by suppressing appetite [13]. The primary effects of leptin on energy balance arise from its capacity to decrease food intake and induce thermogenesis [14]. In individuals who have sufficient nutrition, the concentration of leptin in the blood plasma is directly connected to the amount of body fat they have. Hence, leptin serves as a messenger for the quantity of stored energy in the form of fat tissue, thereby controlling the intake and expenditure of energy to uphold body weight [15]. Obese people have greater levels of serum leptin due to reduced sensitivity to leptin. Leptin levels in the blood are strongly associated with the percentage of body fat and decrease when weight is lost [16].

Asprosin is a recently identified hormone that has been shown to increase appetite and stimulate the liver to produce glucose during fasting. Both obese humans and mice have been observed to have higher than normal levels of this hormone in their circulation [17,18]. Asprosin is released by white adipose tissue, circulates at low concentrations, and is taken in by the liver. In the liver, it stimulates the G protein-cAMP-PKA pathway, leading to the quick release of glucose into the circulation [17].

Obesity is a persistent inflammatory state that results in oxidative damage. Oxidative stress occurs when there is an unequal amount of reactive oxygen species (ROS) compared to the cell’s antioxidant defense mechanism. ROS, which are increased in obesity, exerts their effects on hypothalamic neurons to regulate appetite and fullness, ultimately controlling body weight [19]. The exact mechanism by which obesity induces oxidative stress is not completely understood. However, research has shown that being overweight increases TBARS levels through lipid peroxidation and decreases antioxidant enzymes by decreasing total thiol levels in tissues [20,21]. The cellular antioxidant defense systems and dietary antioxidants neutralize the harmful effects of free radicals [19]. White tea (WT) has been shown to reduce MDA levels and increase glutathione and antioxidant enzyme levels due to its catechin and flavonoid content, thereby reducing oxidative stress [22,23]. As a result, the utilization of low-cost natural chemicals possessing few side effects and potent antioxidant properties, such as teas, can mitigate the negative consequences associated with obesity [24].

Tea is one of the most widely drank beverages globally, second only to water. Theaceae plant species *Camellia sinensis*, grown in more than 30 nations worldwide, produces tea by steeping its leaves. WT is made from the tender leaves or buds of tea plants that are covered in fine, silver-colored hairs. These leaves are gathered during the beginning of spring. It is more uncommon and undergoes less processing compared to other varieties of tea. To minimize chlorophyll production, the buds may be protected from sunlight while growing, resulting in the young leaves appearing white [25]. WT is subjected to prolonged withering and drying processes without oxidation and enzyme deactivation [26]. Minimal processing of WT results in a higher concentration of polyphenol phytochemicals, including catechins [27]. The main catechin in WT is EGCG, considered tea’s most bioactive component [28]. As flavanols are present in high concentrations in the tea plant (*Camellia sinensis*), tea consumption represents a significant source of these flavonoids [29]. WT offers numerous health benefits owing to its potent antioxidant qualities and significant tea components that play an important role in disease prevention. Furthermore, WT can serve as a beneficial adjunct or substitute therapy for combating obesity and its associated consequences [30]. Previous studies have shown that white tea, with its high antioxidant content, has potential healing effects on various diseases [31,32,33,34]. Therefore, with the regular addition of white tea to the daily diet, antioxidant consumption will increase, and protection against various diseases will be provided.

According to reports, WT has been shown to reduce MDA levels and increase glutathione and antioxidant enzyme levels due to its catechin and flavonoid content, thereby reducing oxidative stress [22,23].

Although the benefits of tea are mainly attributed to catechins, some research suggests that synergism between all tea components is therapeutically more effective than some of the components consumed alone [28]. Furthermore, catechins and caffeine have been shown to be synergistic in anti-obesity activities [35]. This is why we used the plant containing all components, not only catechins, in our study.

The objective of this study was to examine the weight-reducing properties of WT derived from the *Camellia sinensis* plant, which is cultivated in Türkiye. This was achieved by analyzing various biochemical factors such as serum leptin, asprosin, lipids, AST and ALT enzymes, fasting blood glucose, insulin levels, HOMA-IR index, oxidative stress markers, and the number and size of adipocytes in adipose tissue through a histopathological examination.

## 2. Materials and Methods

### 2.1. Animals Studies, Preparation of White Tea, Experimental Design

Seventy-two male Sprague–Dawley rats (6–8 week-old male, 250–300 g) were obtained from the Animals Research Center of Recep Tayyip Erdogan University Laboratory. Ethical approval for this study was obtained from Recep Tayyip Erdogan University’s Laboratory Animals Local Ethics Committee (Decision number: 2020-24/26 June 2020). All rats were kept under controlled temperature (22 °C ± 2 °C) and humidity (55% ± 5%) in a 12 h light/dark cycle, with free access to water and standard rat chow. After 1 week of adaptation, all animals were fed a standard rat chow or a high-fat diet (HFD, 44% of energy from fat, Arden Research & Experiment) and divided into nine groups of 8 animals per group. Orlistat (ORL, Thincal, Kocak Farma Ilac ve Kimya Sanayi, Istanbul, Türkiye) was selected as the positive control in this study. The dosage of orlistat in our study was established at 30 mg/kg [36]. In order to evaluate the protective and therapeutic effects separately, rats were divided into two main groups as protective and therapeutic groups.

In order to evaluate the protective effect, rats were randomly divided into 4 groups as control, high-fat diet (HFD), high-fat diet + white tea (HFD+WT), and high-fat diet + orlistat (HFD+ORL). Tap water, 5 mg/kg of WT, or 30 mg/kg of ORL was given daily by oral gavage for 12 weeks. The control and HFD groups were given tap water 2 days a week by oral gavage for 12 weeks.

To evaluate the therapeutic effect, the rats were randomly divided into 5 groups: obesity (OB), obesity + orlistat (OB+ORL), obesity + 50 mg/kg white tea (OB+WT50), and obesity + 150 mg/kg white tea (OB+WT150). After the obesity criteria were met, the therapeutic effect of WT was investigated. For the determination of the therapeutic effect, rats were fed a high-fat diet for 12 weeks, followed by 4 weeks of standard rat chow. Rats fed with standard rat chow for the last 4 weeks were given 50 mg/kg WT, 150 mg/kg WT, and 30 mg/kg ORL. The control and HFD groups were given tap water 2 days a week by oral gavage for 4 weeks.

WT leaves were obtained from the manufacturer (General Director of Tea Enterprises, Rize, Türkiye). The WT leaves used were from harvested a *Camellia sinensis* plant in May 2020. The brewing method used for white tea was previously reported [37]. The tea leaves were weighed in the amounts calculated for the studies, added to water boiled at 100 °C and slightly rested (97–98 °C), and covered and infused for 10 min. This temperature and brewing time were chosen because of the high antioxidant content in white tea [37]. WT was stored in a dark and humidity-free environment under appropriate conditions during the study. In addition, since WT was prepared daily, there was no loss of antioxidants, and it was kept at room temperature and given to rats. Body weights were recorded once a week. The doses to be administered to the rats were recalculated. As an obesity criterion, the weight gain of the rats in the case groups was determined by 20% compared to the control group [38,39]. In the group in which the protective effect of white tea was examined, the study was terminated when the weight of the rats given a high-fat diet was 20% higher than the control group. When the weights of the rats in the groups in which the therapeutic effect of white tea was examined were 20% higher than the control group, the groups were separated to be given white tea and orlistat. At this stage, after 12 h of fasting, the rats in each group were anesthetized with a combination of ketamine (100 mg/kg, i.p.) and Rompun (10 mg/kg, i.p.), and blood was taken from the aorta, transferred without anticoagulant tubes, and kept at room temperature for one hour to allow fibrin to form. The samples were then centrifuged at 3500 rpm for 15 min at 2–8 °C. The obtained supernatants were stored at −80 °C until the biochemical parameters were studied. Retroperitoneal adipose tissues were collected from rats for study. The adipose tissues of rats that were sacrificed under anesthesia were preserved in a 10% formaldehyde solution for histopathological and immunohistochemical analyses.

### 2.2. White Tea Composition

The composition of WT is shown in Table 1.

### 2.3. Biochemical Analysis

The leptin, asprosin, and insulin levels were determined in serum samples using ELISA (The Bioassay Technology Laboratory Rat Kits, catalog no: E0561Ra, E1703Ra, E0707Ra) (Shanghai, China) following the manufacturer’s procedure. Thiobarbituric acid reactive substances (TBARS) were formed by modifying the Draper and Hadley method [40]. The Total Thiol (TT) groups were performed using Ellman’s reagent [41]. Serum glucose, triglyceride (TG), total cholesterol (TC), low-density cholesterol (LDL-C), high-density lipoprotein cholesterol (HDL-C), aspartate aminotransferase (AST), and alanine aminotransferase (ALT) levels were analyzed with the assistance of an autoanalyzer (Beckman Coulter AU5800). Insulin resistance, HOMA-IR (HOMA-IR = [Fasting blood glucose (mg/dL) × Fasting insulin (μU/mL)]/405), was calculated [42].

### 2.4. Histopathological Analysis

#### 2.4.1. Histology Tissue-Tracking Processing

Adipose tissue sections removed from the rats were trimmed to a volume of 1.5 cm^3^. They were then fixed in 10% formalin solution for 24–36 h, placed in tissue-tracking cassettes, and subjected to routine histologic tissue tracking in a tissue-tracking device (Thermo Scientific™ Citadel 2000, Cheshire, UK). After tissue tracking, adipose tissue samples were blocked with hard paraffin (Merck GmbH, Darmstadt, Germany) using a paraffin-blocking device (Leica EG 1150 H, Leica Biosystems Inc., Leica Biosystems Nussloch GmbH, Heidelberger Str. 17-19 D-69226 Nussloch, Germany). Adipose tissue paraffin blocks were serially sectioned at 4–5 μm thickness using a rotary microtome (Leica RM2255, Leica Biosystems Nussloch GmbH, Heidelberger Str. 17-19 D-69226 Nussloch, Germany) and placed on adhesive and positively charged slides (Superior Marinfield Histobond+).

#### 2.4.2. Hematoxylin and Eosin (H&E) Staining Method

Adipose tissue sections were stained with H&E using a staining device (Leica ST5020, Lecia Biosystems, Germany).

#### 2.4.3. Cryostat Sectioning Method

Adipose tissue samples removed from subjects were trimmed to 1.5 cm^3^ volume. Subsequently, adipose tissue samples were kept at −80 °C overnight and sectioned at −14 °C using histopathologist’s freezing medium (Sigma-Aldrich, Darmstadt, Germany) at 6–7 µm thickness with a cryostat (Leica 3050S, Leica Biosystems Nussloch GmbH, Heidelberger Str. 17-19 D-69226 Nussloch, Germany). According to the manufacturer’s catalog, adipose tissue sections were stained with Oil O red (Merck Millipore, 102419, Darmstadt, Germany). Adipose tissue sections stained with Oil O red were then counterstained with Mayers hematoxylin (Merck KGAa, Darmstadt, Germany).

#### 2.4.4. Semi-Quantitative Analysis

The H&E-stained sections were scored for histopathologic damage by assessing the presence of hypertrophic adipocytes, edematous areas, infiltrative areas, and vascular congestion following the findings of previous studies on the histopathologic effects of a high-fat diet on adipose tissue, as shown in Table 2 [43,44,45,46]. In each rat preparation, 20 different areas were scored by two different histopathologists who were blinded to the study groups.

#### 2.4.5. Quantitative Analysis

**Figure 1:**
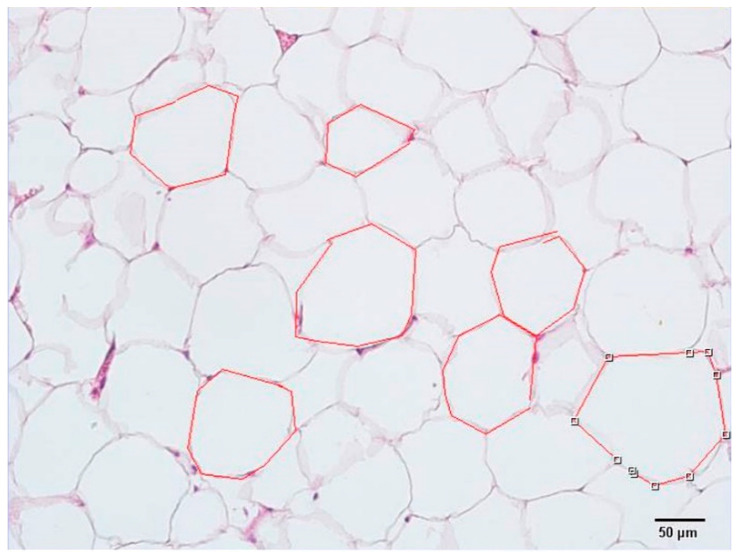
Quantitative analysis: representative light microscopic screenshot of the measurement of the superficial area of adipocytes.

Adipose tissue sections were examined under a light microscope (Olympus BX51, Tokyo, Japan) with a digital attachment (Olympus DP71, Tokyo, Japan), and the superficial area was calculated using the polyline probe of the Olympus DP2.SW computer program as shown (Figure 1).

#### 2.4.6. Stereological Analysis

Nucleated cells were counted using a numerical density measurement in mm^3^ of a Stereo Investigator software system (MicroBrightField 9.0, Colchester, VT, CA, USA). IHC-stained specimens were analyzed using stereological analysis software to determine the areas to be counted following the histopathologist’s preliminary study [47]. Counting counter frames (counting frames) were randomly distributed in the Stereo Investigator program in adipose tissue sections. IHC-positive cells were counted in 35 counting frames in each preparation [48]

### 2.5. Statistical Analysis

All data were analyzed using SPSS 18.0 (IBM, Armonk, NJ, USA) software. For biochemical analyses, intergroup comparisons of the data appropriate to the normal distribution were performed via a one-way analysis of variance (one-way ANOVA) and LSD’s post hoc test. The results of the analyses were given as the mean (X) and standard deviation (SD). A Kruskal–Wallis test was performed for the data that did not show normal distribution, followed by a Mann–Whitney U test as a post hoc test to determine which groups differed. The values obtained were expressed as the median (interquartile range). *p  <*  0.05 was considered statistically significant.

The non-parametric data obtained from semi-quantitative histopathological analyses were calculated as the median and 25% and 75% interquartile ranges, considering maximum and minimum values. Differences between groups were analyzed using a non-parametric Kruskal–Wallis test, followed by a Tamhane T2 test, after which the numerical data of the groups were analyzed (*p*-value < 0.05 was considered statistically significant). Parametric data obtained by quantitative analyses were calculated as arithmetic mean and standard deviation values. Differences between groups were analyzed using a one-way ANOVA test followed by a Tukey HSD test (*p*-value < 0.05 was considered statistically significant).

## 3. Results

### 3.1. Biochemical Results of the Study to Evaluate the Protective Effect of White Tea

**Figure 2:**
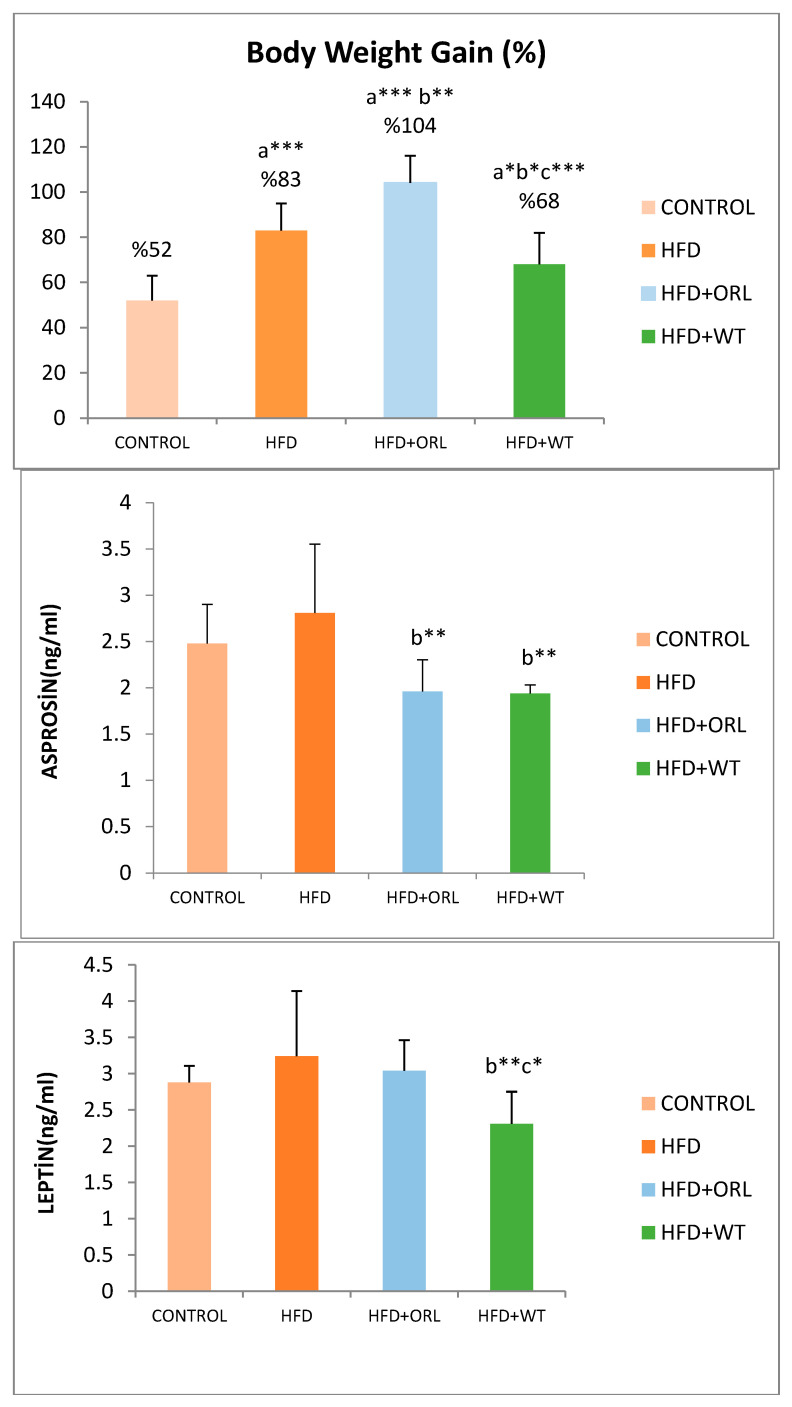
Body weight gain, serum leptin, and asprosin levels of the groups. *: p < 0.05, **: *p* < 0.01, ***: *p* < 0.001. a: Compared to control group. b: Compared to high-fat diet group. c: Compared to the HFD+ORL group. ANOVA/LSD Test.

The effects of WT on biochemical parameters in rats are shown in Table 3. The effects of WT on body weight in rats are shown in Figure 2. Weight gain was significantly lower in rats fed a high-fat diet and given white tea (HFD+WT) for 12 weeks. However, rats fed only a HFD showed a significant increase in body weight compared to the control group (*p* < 0.05) (Figure 2). WT decreased serum leptin, asprosin, glucose, TC, LDL-C, and TBARS levels, as well as HOMA-IR. However, WT was found to significantly increase TT levels in rats fed a high-fat diet (*p* < 0.05) (Table 3).

### 3.2. Biochemical Results of the Study to Evaluate the Therapeutic Effect of White Tea

**Figure 3:**
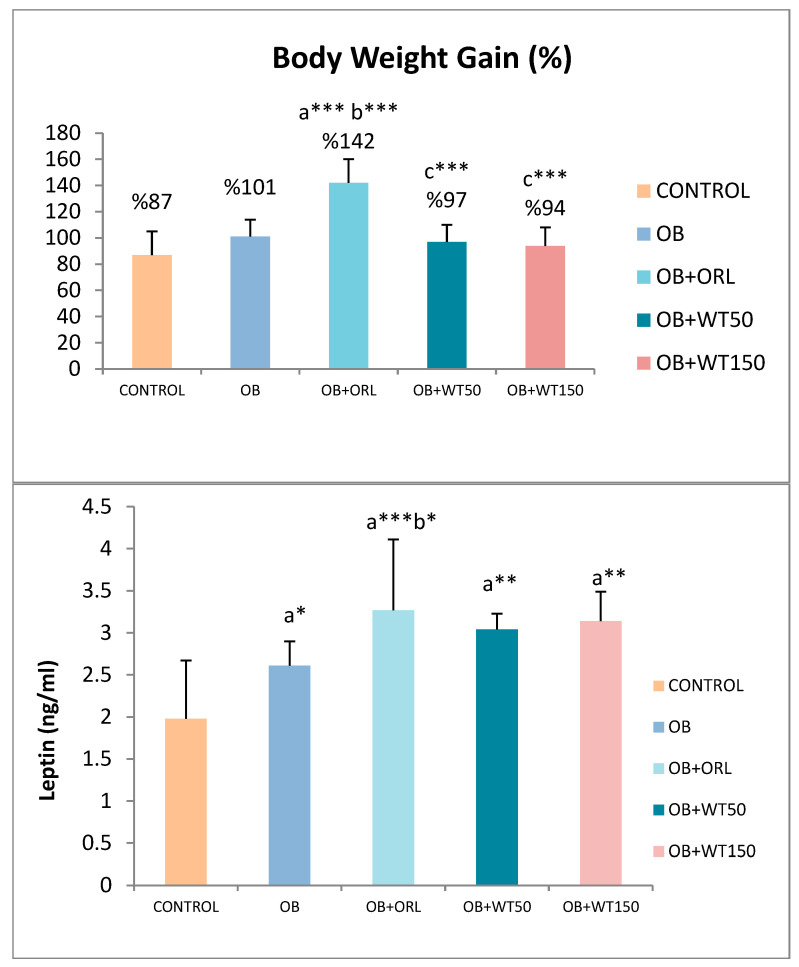

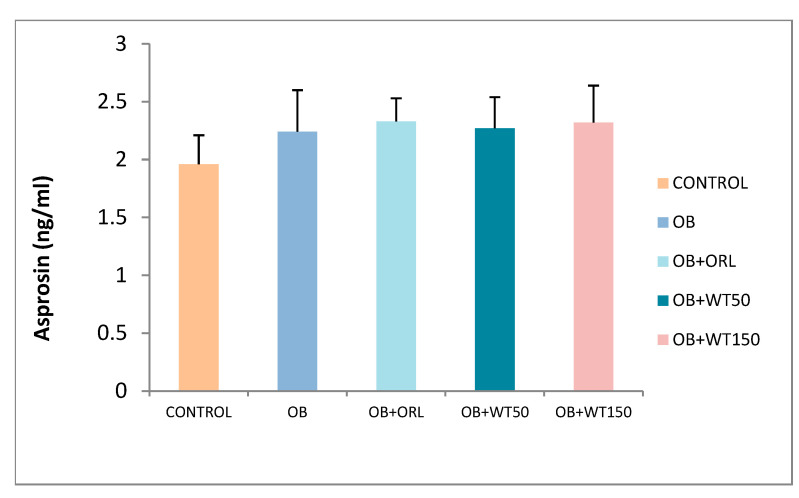
Body weight gain, serum leptin, and asprosin levels of the groups. *: *p* < 0.05, **: *p* < 0.01, ***: *p* < 0.001. a: Compared to the control group. b: Compared to OB group. c: Compared to OB+ORL group. ANOVA/LSD Test.

The effects of WT on biochemical parameters in rats are shown in Table 4. The effects of WT on body weight in rats are shown in Figure 3.

### 3.3. Histopathological Results of the Study to Evaluate the Protective Effect of White Tea

**Figure 4:**
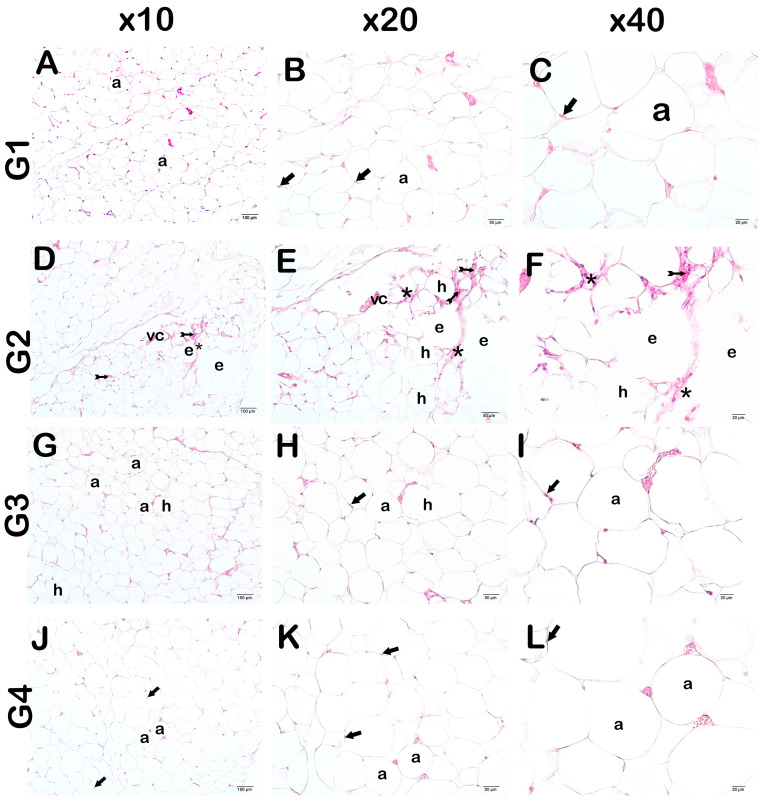
Representative light microscopic image of adipose tissue stained with Harris Hematoxylin and Eosin G. Adipocyte (a), Nucleus (arrow). (**A**) (×10)-(**B**) (×20)-(**C**) (×40): Adipocytes with normal nuclei (arrow) are observed in adipose tissue sections of the control group (G1) (HPS: 0 (0–0)). (**D**) (×10)-(**E**) (×20)-(**F**) (×40): Hypertrophic adipocytes (h), which are large and circular in appearance, are commonly observed in the sections of adipose tissue of the HFD group (G2). In addition, an increase in extracellular matrix (asterisks), edema (e), diffuse inflammation (arrow with tail), and vascular congestion (vc) are observed (HPS: 2 (1.5–2)). (**G**) (×10)-(**H**) (×20)-(**I**) (×40): It is observed that the number of hypertrophic adipocytes decreased in the adipose tissue sections of the HFD+ORL group. There was also a decrease in vascular congestion and infiltrative cells (HPS: 0 (0–1)). (**J**) (×10)-(**K**) (×20)-(**L**) (×40): Although a decrease in hypertrophic adipocytes is observed in the HFD+WT group, typical structured adipocytes (arrow) are widely observed. In addition, vascular congestion and inflammation were decreased (HPS: 0 (0–1)).

**Figure 5:**
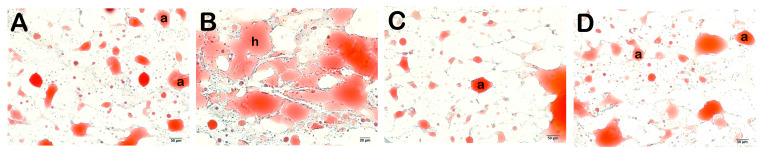
Representative light microscopic image of cryostat sections of adipose tissue stained with Olive O Red. Adipocyte (a) (**A**) (×20): Sections of adipose tissue of the control group (G1) showing normal adipocytes (a) (HPS: 0 (0–0)). (**B**) (×20): Hypertrophic adipocytes (h) with large and circular appearance are commonly observed in the adipose tissue sections of the HFD group (G2) (HPS: 2 (1.5–2)). (**C**) (×20): A decrease in the number of hypertrophic adipocytes was observed in the adipose tissue sections of the HFD+ORL group (HPS: 0 (0–1)). (**D**) (×20): Although there is a decrease in hypertrophic adipocytes in the HFD+WT group, typical adipocytes (a) are commonly observed (HPS: 0 (0–1)).

In adipose tissue sections examined under light microscopy, the control group had adipocytes with a normal nucleus content [Figure 4A–C, Table 5, HPS:0 (0–0)]. In contrast, in the adipose tissue sections of the HFD group, there were widespread large and circular hypertrophic adipocytes and edematous areas. Additionally, there were infiltrative areas and vascular congestion [Figure 4D–F and Figure 5A, Table 5, HPS:2 (1.5–2)]. In the adipose tissue sections of the orlistat-treated group, we observed a decrease in hypertrophic adipocytes, edematous areas, infiltrative cells, and vascular congestion [Figure 4G–I and Figure 5B, Table 5, HPS:0 (0–1)]. In the sections of the WT group, there were typical structured adipocytes, along with a decrease in hypertrophic adipocytes and edematous areas. In addition, we found that infiltrative areas and vascular congestion were reduced [Figure 4J–L and Figure 5C, Table 5, HPS:0 (0–1)].

#### 3.3.1. Semi-Quantitative Findings

In accordance with studies that histopathologically addressed the effects of a high-fat diet on adipose tissue, when the histopathological damage score (HPS) was calculated by taking into account hypertrophic adipocytes, edematous areas, infiltrative areas, and vascular congestion, we found that the HPS score, which was 0 (0–0) in the control group, increased to 2 (1.5–2) in the HFD group [Figure 4A–F and Figure 5A,B, Table 5, *p* = 0.000; HPS:2 (1.5–2)]. On the contrary, we observed that the HPS score, which was measured as 2 (1.5–2) in the HFD group, decreased to 0 (0–1) in the HFD+ORL group [Figure 4D–I and Figure 5B,C, Table 5, *p* = 0.000; HPS:0 (0–1)]. Similarly, we found that the HPS score, which was measured as 2 (1.5–2) in the HFD group, decreased to 0 (0–1) in the HFD+WT group. [Figure 4D–F,J–L and Figure 5B–D, Table 5, *p =* 0.000; HPS:0 (0–1)].

#### 3.3.2. Quantitative Findings

When we measured the surface areas of adipocytes, we found that the average surface area of adipocytes measured as 384.91 ± 22.61 µm^2^ in the control group increased to 479.62 ± 19.12 µm^2^ in the HFD group (Figure 5A,B, Table 6; *p* = 0.000). On the contrary, we observed that the mean surface area of adipocytes, which was measured as 479.62 ± 19.12 µm^2^ in the HFD group, decreased to 393.91 ± 18.81 µm^2^ in the HFD+ORL group (Figure 5B,C, Table 6; *p =* 0.000). Similarly, we observed that the mean surface area of adipocytes, which was measured as 479.62 ± 19.12 µm^2^ in the HFD group, decreased to 401.43 ± 50.71 µm^2^ in the HFD+WT group (Figure 5B–D Table 6; *p =* 0.000).

#### 3.3.3. Stereological Analysis

We found that the numerical density of adipocytes in the control group was 12.65 ± 2.93 µm^3^, while it increased to 32.25 ± 4.59 µm^3^ in the HFD group (Table 7, *p* = 0.000). On the contrary, we observed that the numerical density, which was measured as 32.25 ± 4.59 µm^3^ in the HFD group, decreased to 24.00 ± 4.65 µm^3^ in the HFD+ORL group (Table 7, *p* = 0.000). Similarly, we found that the numerical density, which was measured as 32.25 ± 4.59 µm^3^ in the HFD group, decreased to 17.55 ± 2.74 µm^3^ in the HFD+WT group (Table 7, *p* = 0.000).

### 3.4. Histopathological Results of the Study to Evaluate the Therapeutic Effect of White Tea

**Figure 6:**
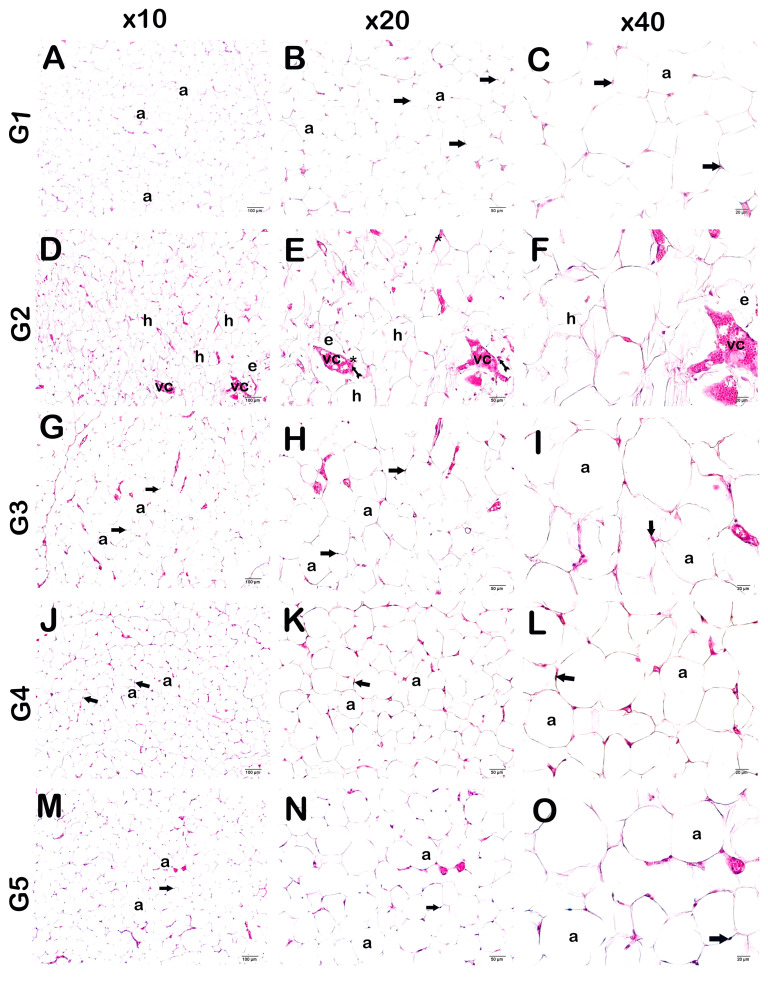
Representative light microscopic image of adipose tissue stained with Harris hematoxylin and eosin G. Adipocyte (a), Nucleus (arrow). (**A**) (×10)-(**B**) (×20)-(**C**) (×40): Adipocytes with normal nucleus (arrow) are observed in adipose tissue sections of the control group (G1) (HPS: 0 (0–0)). (**D**) (×10)-(**E**) (×20)-(**F**) (×40): In adipose tissue sections of the OB group (G2), large and circular hypertrophic adipocytes (h) are commonly observed. In addition, an increase in extracellular matrix (asterisks), edema (e), diffuse inflammation (arrow with tail), and vascular congestion (vc) are observed (HPS: 2 (1.5–2)). (**G**) (×10)-(**H**) (×20)-(**I**) (×40): A decrease in the number of hypertrophic adipocytes was observed in the adipose tissue sections of the OB+ORL group. There is also a decrease in vascular congestion and infiltrative cells (HPS: 0 (0–1)). (**J**) (×10)-(**K**) (×20)-(**L**) (×40): A decrease in hypertrophic adipocytes was observed in the OB+WT50 treatment group. In addition, vascular congestion and inflammation were decreased (HPS: 0 (0–1)). (**M**) (×10)-(**N**) (×20)-(**O**) (×40): In the OB+WT150 treatment group, hypertrophic adipocytes, vascular congestion, and inflammation were decreased, and typical adipocytes were commonly observed (HPS: 0 (0–1)).

**Figure 7:**
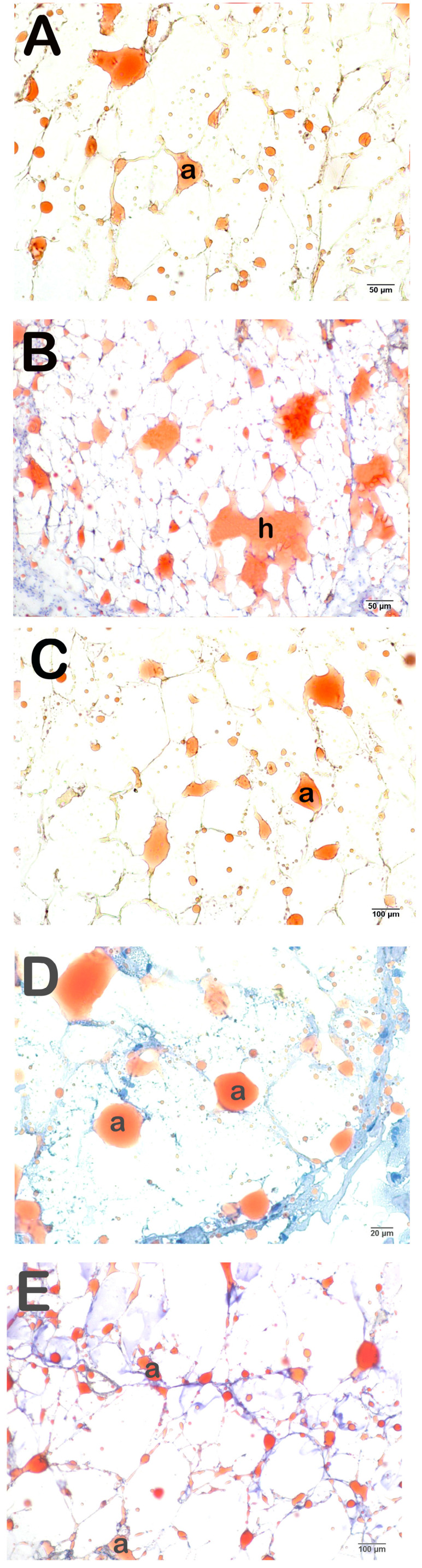
Representative light microscopic image of cryostat sections of adipose tissue stained with Olive O Red. Adipocyte (a) (**A**) (×20): Sections of adipose tissue of the control group (G1) showing normal adipocytes (a) (HPS: 0 (0–0)). (**B**) (×20): In adipose tissue sections of the OB group (G2), large and circular hypertrophic adipocytes (h) are commonly observed (HPS: 2 (1.5–2)). (**C**) (×20): A decrease in the number of hypertrophic adipocytes was observed in the adipose tissue sections of the OB+ORL group (HPS: 0 (0–1)). (**D**) (×20): In the OB+WT50 treatment group, hypertrophic adipocytes were decreased, and typical adipocytes (a) were commonly observed (HPS: 0 (0–1)). (**E**) (×20): Although hypertrophic adipocytes were decreased in the OB+WT150 treatment group, typical adipocytes (a) were commonly observed (HPS: 0 (0–1)).

In adipose tissue sections examined under light microscopy, control tissue had adipocytes with a normal nucleus content [Figure 6A–C, Table 8, HPS:0 (0–0)]. In contrast, adipose tissue sections from the OB group showed diffuse large and circular hypertrophic adipocytes and oedematous areas, as well as infiltrative areas and vascular congestion [Figure 6D–F and Figure 7B, Table 8, HPS:2 (1.5–2)]. In the adipose tissue sections of the OB+ORL group, we observed a decrease in hypertrophic adipocytes, edematous areas, infiltrative cells, and vascular congestion [Figure 6G–I and Figure 7C, Table 8, HPS:0 (0–1)]. In the sections of the OB+WT50 and OB+WT150 groups, in addition to the decrease in hypertrophic adipocytes and edematous areas, there were also adipocytes with typical structure. In addition, we found that infiltrative areas and vascular congestion were reduced [Figure 6J–O, Figure 7D,E, Table 8, respectively, HPS:0 (0–1); 0 (0–1)].

#### 3.4.1. Semi-Quantitative Findings

In accordance with studies that histopathologically addressed the effects of a high-fat diet on adipose tissue, we found that the histopathological damage score (HPS), calculated by taking into account hypertrophic adipocytes, edematous areas, infiltrative areas, and vascular congestion, was 0 (0–0) in the control group, while it increased to 2 (1.5–2) in the OB group [Figure 6A–F and Figure 7A,B, Table 8, *p* = 0.000; HPS:2 (1.5–2)]. On the contrary, we observed that the HPS score, which was measured as 2 (1.5–2) in the OB group, decreased to 0 (0–1) in the OB+ORL group [Figure 6D–I and Figure 7B,C, Table 8, *p* = 0.000; HPS:0 (0–1)]. Similarly, we observed that the HPS score, which was measured as 2 (1.5–2) in the OB group, decreased to 0 (0–1) in the OB+WT50 group [Figure 6D–F,J–L and Figure 7B–D, Table 8, *p* = 0.000; HPS:0 (0–1)].

#### 3.4.2. Quantitative Findings

When we measured the superficial areas of adipocytes, we found that the average surface area measured as 383.81 ± 21.62 µm^2^ in the control group increased to 477.41 ± 17.10 µm^2^ in the OB group (Figure 7A,B, Table 9; *p* = 0.001). In contrast, we observed that the mean superficial area measured as 477.41 ± 17.10 µm^2^ in the OB group decreased to 395.77 ± 16.54 µm^2^ in the OB+ORL group (Figure 5B,C, Table 9; *p* = 0.001). Similarly, we observed that the mean superficial area measured as 477.41 ± 17.10 µm^2^ in the HFD group decreased to 402.85 ± 49.36 µm^2^ in the 0B+WT50 group and to 399.74 ± 49.01 µm^2^ in the OB+WT150 group (Figure 7B–D, Table 9; *p* = 0.001).

#### 3.4.3. Stereological Analysis

As a result of stereological measurements of adipocytes in fat tissue with a nucleator probe in an optical dissector using the StereoInvestigator (9.0 MBF, USA) program, we measured the adipocyte numerical density in the control group as 1.74 ± 0.61 per µm^3^. We observed that it increased to 4.17 ± 0.82 µm^3^ in the OB group (Table 10, *p* = 0.000). Compared to the OB group, we found that adipocyte numerical density decreased to 2.06 ± 0.91 µm^3^ in the OB+ORL group (Table 10, *p =* 0.000). Similarly, we observed that the adipocyte numerical density measured as 4.17 ± 0.82 µm^3^ in the OB group decreased to 1.76 ± 0.62 µm^3^ in the OB+WT50 group (Table 10, *p =* 0.000), and the adipocyte numerical density measured as 4.17 ± 0.82 µm^3^ in the OB group decreased to 1.35 ± 0.54 µm^3^ in the OB+WT150 group (Table 10, *p =* 0.000).

## 4. Discussion

Obesity is characterized by an accumulation of excessive fat mass and the enlargement of adipose tissue, resulting from both the increase in size and number of fat cells. This condition is influenced by various variables, including lifestyle choices, behaviors, environmental factors, and genetic predisposition [1]. WT has a higher content of total polyphenols and catechins than other teas and has a soft and sweet taste, unlike the taste of green tea. WT is therefore becoming an increasingly popular beverage [49,50].

This study aimed to examine the possible weight-reducing properties of WT derived from the *Camellia sinensis* plant on Sprague–Dawley rats who were fed a high-fat diet. Our study revealed that WT possesses an anti-obesity property, as it effectively decreases the weight of rats that were fed a high-fat diet. Giving WT in combination with a high-fat diet successfully reduced body weight as well as serum levels of leptin, asprosin, lipids, fasting blood glucose, insulin, TBARS, and HOMA-IR. It successfully improved histologic observations in adipose tissue, including the number and size of adipocytes.

Obesity, a chronic inflammatory condition, is characterized by increased levels of leptin in the blood, which is associated with complications [51]. Our research has shown that feeding a high-fat diet results in increased leptin levels in the circulation. Certain micronutrients, macronutrients, and bioactive dietary compounds have the capacity to enhance leptin sensitivity and counteract leptin resistance in individuals with obesity [51]. EGCG has been demonstrated to decrease food consumption and blood leptin resistance while promoting energy expenditure and fat oxidation, based on in vivo evidence [52]. In previous studies, green tea consumption in mice fed a high-fat diet significantly decreased the weight of their body fat tissues and their leptin resistance [53,54]. WT has higher total polyphenol and catechin concentrations compared to other teas [49]. WT is characterized by its exceptional antioxidant potential. A study evaluating the antioxidant properties of various plant species, including green tea, in vivo and in vitro, confirmed that WT had the highest antioxidant capacity [55]. Since tea polyphenols can improve leptin sensitivity by modulating obesity-related inflammatory processes and providing a weight-reducing effect, we suggest that white tea containing high levels of catechins and polyphenols may be effective in reducing serum leptin resistance [56,57]. In our study, similar to the literature, it was observed that weight gain was significantly lower in rats fed a high-fat diet for 12 weeks with white tea administration (HFD+WT). In the same group, white tea was observed to reduce leptin resistance. In addition, a significant increase in body weight was observed in rats fed with HDF compared to the control group. Considering these results, the potential relationship between white tea and leptin may contribute to the management of obesity.

Recent investigations have indicated that adipocytes in the adipose tissue of obese rats experience alterations in both their form and quantity. In obesity-focused research on rats, a high-fat diet leads to an increase in the size of adipocytes and an overall increase in the number of cells in adipose tissue [58]. Similarly, another study has shown in animal model experiments that adipocytes are enlarged, indicating hypertrophy [45]. Furthermore, in another study, they observed large areas of infiltrating cells, vascular congestion, and also hypertrophic adipocytes [43]. In another study, they observed that the obesity model developed through a high-fat diet in rats had a wide distribution of hypertrophic adipocytes, infiltrative areas, and edematous areas [44]. Similarly, in our study, we observed that the number of hypertrophic adipocytes increased, and there were oedematous areas accompanied by diffuse infiltrative areas and vascular congestion in the high-fat diet groups. Conversely, we determined that WT had ameliorative effects on these findings.

Our investigation reported that feeding a high-fat diet increased serum asprosin levels. Given the antioxidant and anti-inflammatory characteristics of WT, we hypothesized that it could potentially help reduce the negative effects of asprosin-induced metabolic dysfunction. The present study found that the consumption of WT led to a notable reduction in asprosin levels in rats that were fed a high-fat diet. High levels of asprosin in the circulation have been linked to conditions related to excessive nutrition, such as obesity, type 2 diabetes, and insulin resistance [59,60,61]. There was a significant positive correlation between serum asprosin levels and hyperglycemic, lipidemic, and proinflammatory conditions [62]. A clinical investigation showed a notable reduction in asprosin levels following weight loss [63]. The potential impact of WT on asprosin levels is a novel area of research.

The diet is a potential component that contributes to the production of ROS during the development of obesity and its related risk factors [64]. High-fat diets can potentially modify oxygen metabolism. Obese individuals who consume high-fat diets experience notable oxidative damage and inflammation. Low antioxidant defense is a contributing cause of oxidative stress in obesity. Insufficient consumption of antioxidant-rich phytochemicals may result in inadequate defense against oxidative stress [65]. In our study, WT demonstrated support for these findings by reducing the levels of TBARS, a marker of oxidative cell damage caused by a high-fat diet. In addition, we observed a decrease in serum TT levels in rats fed a high-fat diet, while the WT group had an increase in TT levels. Based on these findings, we concluded that WT had a beneficial impact on the lipid peroxidation caused by a high-fat diet. The results of our research were corroborated by previous studies showing that WT effectively reduced MDA levels in adipose tissue and liver [22,23,66].

WT effectively inhibits adipogenesis and stimulates lipolysis activity in human preadipocytes in vitro and is a natural source with anti-obesity effects that can modulate the adipocyte life cycle at different stages [67]. A study has shown that WT has a better inhibition capacity against lipase activity compared to green and black teas. In this study, the results confirmed that the capacity of tea extracts to inhibit triacylglycerol hydrolase activity in vitro was significantly correlated with their flavan-3-ol (catechin) and EGCG contents [68]. Our study showed that administration of EGCG-rich WT with a high-fat diet significantly prevented weight gain compared to the group fed only a high-fat diet. These results indicated that WT consumption is effective in preventing an increase in body weight. Similarly, another study showed that WT has a significant effect on decelerating weight gain [69]. Previous animal studies have also provided evidence for the substantial impact of WT in preventing weight gain [22,70]. In another study, *Camellia sinensis* tea species decreased body weight and white fat accumulation in high-fat-diet-induced obese mice. Among the other tea types, WT exhibited the most effective anti-obesity effect [71].

Orlistat is an FDA-approved drug for the long-term treatment of obesity. Orlistat exerts its effects specifically on gastrointestinal lipase by inhibiting the hydrolysis of dietary fat intake into absorbable free fatty acids and glycerol [72]. Our investigation revealed that body weights were higher in the group given orlistat compared to the other groups. In our work, we hypothesize that orlistat, a drug that reduces fat absorption, may increase appetite in rats with unrestricted access to food. This effect is believed to be related to a loss in satiety, leading to greater weight gain.

Obesity is closely linked to insulin resistance and high blood glucose levels. The consumption of a diet high in fat has an impact on the way glucose and lipids are metabolized in the body. This leads to a decline in the functioning of the liver and other important metabolic organs, resulting in a gradual increase in hyperglycemia, hyperinsulinemia, and the development of insulin resistance over time [65]. A previous study reported that WT has significant antioxidant activity and can help alleviate oxidative stress and insulin resistance, hence exerting anti-diabetic effects [73]. Another study showed that when rats on a high-fat diet were given WT at the same time, their fasting blood insulin levels and HOMA-IR were lower compared with rats on a high-fat diet alone [22]. Our study found that when rats on a high-fat diet were given WT, there was a notable reduction in serum glucose levels and HOMA-IR. These findings indicate that WT has antidiabetic properties in rats that were given a high-fat diet.

Hyperlipidemia is a lipid metabolism abnormality characterized by an increase in total cholesterol, triglyceride, and LDL-C and/or a decrease in HDL-C in the circulation. The cholesterol-lowering effects of edible plants have been extensively researched, and several natural products have demonstrated their ability to reduce plasma cholesterol levels [74]. The results of our study revealed that rats fed a high-fat diet exhibited elevated levels of total cholesterol, triglyceride, HDL-C, and LDL-C compared with the other groups. However, these levels were reduced by WT administration in combination with a high-fat diet. Notably, the decrease in LDL-C and total cholesterol levels was statistically significant. The present study demonstrated that WT exhibited a hypolipidemic effect in rats on a high-fat diet. It also revealed that orlistat led to a statistically significant decrease in total cholesterol levels [72]. Our results are consistent with previous studies [22,70]. A previous study showed that WT had the highest level of activity in lowering the absorption of cholesterol by HepG2 cells. Additionally, it was reported that WT enhanced the activity of LDL receptor binding [68]. A randomized clinical trial found that the use of WT by patients with type 2 diabetes led to significant reductions in weight, blood glucose, insulin, LDL-C, TC, and TG levels compared to their levels before the experiment and compared to the control group. Additionally, it resulted in improved HDL-C levels [75].

The fact that we did not take blood from all rats before starting the study is a limitation of our study because determining the difference between serum parameters at the beginning and end of the study would have made our study much more meaningful. Our study is a pilot study and focused on oxidative stress and apoptosis with asprosin and leptin hormones. In addition, there is more than one mechanism related to the effect of WT on weight loss, and more studies are needed. Clinical studies are especially insufficient in this field. In order to see the health benefits of WT, further studies are needed on the level of consumption required. These studies can be repeated at different doses and with different study periods. In addition, it is our recommendation to conduct studies in which food intake and fecal monitoring of rats are performed. The effects of WT can be examined in liver tissue. In addition, it is recommended to study asprosin, of which there is a small number of studies in the literature, in various tissues.

This study was conducted to examine the effects of antioxidant-rich white tea on obesity and to make a contribution to the literature in this direction. It would be useful to carry out studies on microbiota in rats, as well as studies in which the food intake, fecal contents, and amounts of rats are monitored. Studies in which white tea is applied to rats at different times and doses will contribute to this field.

## 5. Conclusions

The objective of our study was to investigate the impact of pesticide-free WT on obesity and adipose tissue. Our study found that WT has a preventative effect against obesity by lowering oxidative stress in rats that were fed a high-fat diet. WT may be a potentially beneficial beverage against obesity and its associated complications.

## Figures and Tables

**Table 1:** White tea composition.

White Tea Analysis (HPLC) Results			
	% Dry Matter	**	% Dry Matter
Gallic acid	0.11	Gallic acid	0.18
Caffeine	5.01	Caffeine	4.83
EGC	0.38	EGC	0.00
C	0.00	C	0.06
EC	0.87	EC	0.33
EGCG	9.20	EGCG	4.75
ECG	2.28	ECG	1.07
Total Catechin (EGC+EC+EGCG+ECG)	12.74	Total Catechin (EGC+EC+EGCG+ECG)	6.21

** The infusion process was performed in 10 min by adding 1.5 g of white tea to boiled pure water. The calculations were based on the same standards. ISO14502-2/2005 Standard method was used. It was carried out in ÇAYKUR (General Director of Tea Enterprises) Agricultural Research Laboratory.

**Table 2:** Histopathological damage scoring (HPS).

Score	Result
Hypertrophic Adipocytes	
0	Less than 5%
1	6% to 25%
2	26% to 50%
3	More than 50%
Infiltrative Areas	
0	Less than 5%
1	6% to 25%
2	26% to 50%
3	More than 50%
Oedematous Areas	
0	Less than 5%
1	6% to 25%
2	26% to 50%
3	More than 50%
Vascular Congestion	
0	Less than 5%
1	6% to 25%
2	26% to 50%
3	More than 50%

**Table 3:** Effects of white tea and orlistat on serum parameters.

Parameters	Control (n = 7)	HFD (n = 8)	HFD+ORL (n = 7)	HFD+WT (n = 8)
^1^ Glu (mg/dL)	130 ± 19.3	220 ± 22.6 ^a^***	159 ± 6.86 ^b^**	159 ± 37.46 ^b^***
^2^ Insulin (mIU/L)	4.03 (1.49)	4.93 (0.68) ^a^*	5.24 (0.86) ^a^*	4.84 (0.75) ^c^*
^1^ HOMA-IR	1.5 ± 0.34	2.63 ± 0.33 ^a^***	2.26 ± 0.29 ^a^**	1.97 ± 0.59 ^b^*
^1^ TC (mg/dL)	49 ± 3.61	71 ± 11.72 ^a^***	57 ± 6.71 ^b^**	52 ± 6.08 ^b^***
^1^ HDL-C (mg/dL)	33 ± 1.75	40 ± 1.75 ^a^**	38 ± 5.65	38 ± 5.05
^1^ LDL-C (mg/dL)	9.67 ± 2.73	18.85 ± 6.59 ^a^**	14.57 ± 3.08	13.0 ± 2.37 ^b^*
^1^ TG (mg/dL)	30 ± 5.41	36 ± 12.88	32 ± 7.08	26 ± 4.8
^1^ AST/ALT	3.3 ± 0.72	4.07 ± 0.26 ^a^*	4.53 ± 0.77 ^a^**	3.97 ± 0.47
^1^ Leptin (ng/mL)	2.88 ± 0.226	3.24 ± 0.80	3.04 ± 0.422	2.31 ± 0.44 ^b^**^c^*
^1^ Asprosin (ng/mL)	2.48 ± 0.423	2.81 ± 0.742	1.96 ± 0.344 ^b^**	1.94 + 4 ± 0.092 ^b^**
^2^ TT (mmol/mL)	387 (94)	279 (60) ^a^*	348 (32)	423 (26) ^b^**^c^**
^1^ TBARS (mmol/L)	6.92 ± 0.29	7.83 ± 0.59 ^a^**	6.03 ± 0.78 ^a^**^b^***	5.85 ± 0.33 ^a^**^b^***

*: *p* < 0.05, **: *p* < 0.01, ***: *p* < 0.001. ^a^: Compared to the control group. ^b^: Compared to the HFD group. ^c^: Compared to the HFD+ORL group. ^1^ One-way ANOVA/LSD Test; ^2^ Kruskal–Wallis/Mann–Whitney U Test. Abbreviations: ALT, alanine aminotransferase; AST, aspartate aminotransferase; Glu, glucose; HDL-C, high-density lipoprotein cholesterol; HFD, high-fat diet; HOMA-IR, insulin resistance test; LDL-C, low-density lipoprotein cholesterol; ORL, orlistat; TBARS, thiobarbituric acid reactive substances; TC, total cholesterol; TG, triglyceride; TT, total thiol; WT, white tea.

**Table 4:** Effects of two different doses of white tea and orlistat on serum parameters.

Parameters	Control (n = 7)	OB (n = 7)	OB+ORL (n = 6)	OB+WT50 (n = 6)	OB+WT150 (n = 6)
^1^ Glu (mg/dL)	158 ± 36	179 ± 13	158 ± 46	160 ± 33	198 ± 32
^1^ Insulin (mIU/L)	4.67 ± 0.29	5.34 ± 0.14 ^a^**	5.53 ± 0.25 ^a^**	5.61 ± 0.61 ^a^***	5.56 ± 0.39 ^a^***
^1^ HOMA-IR	2.1 ± 0.58	2.16 ± 0.37	2.2 ± 0.59	2.51 ± 0.75	2.73 ± 0.61
^1^ TC (mg/dL)	67 ± 13.9	90 ± 3.6 ^a^**	79 ± 6.8	79 ± 8.24	70 ± 14.4 ^b^**
^1^ HDL-C (mg/dL)	38 ± 7	43 ± 9.4	47 ± 6.6	46 ± 5.5	40.7 ± 6.7
^1^ LDL-C (mg/dL)	22 ± 6.7	24 ± 6.6	27 ± 4.9	28 ± 3.9	25 ± 7.9
^2^ TG (mg/dL)	39 (12)	44 (19)	42 (24)	31 (14)	36 (18)
^1^ AST/ALT	4.7 ± 0.28	5.35 ± 0.26 ^a^*	4.75 ± 0.7	4.5 ± 0.32 ^b^*	4.22 ± 0.53 ^b^**
^1^ Leptin (ng/mL)	1.98 ± 0.69	2.61 ± 0.29 ^a^*	3.27 ± 0.84 ^a^***^b^*	3.04 ± 0.19 ^a^**	3.14 ± 0.35 ^a^**
^1^ Asprosin (ng/mL)	1.96 ± 0.25	2.24 ± 0.36	2.33 ± 0.20	2.27 ± 0.27	2.32 ± 0.32
^2^ TT (mmol/mL)	310 (170)	305 (59)	339 (75)	361 (43)	333 (137)
^1^ TBARS (mmol/L)	10.1 ± 1.6	11.9 ± 1.39 ^a^*	7.78 ± 0.82 ^a^**^b^***	5.31 ± 0.49 ^a^***^b^***^c^**	6.19 ± 0.98 ^a^***^b^***^c^*

*: *p* < 0.05, **: *p* < 0.01, ***: *p* < 0.001. ^a^: Compared to the control group. ^b^: Compared to the OB group. ^c^: Compared to the OB+ORL group. ^1^ One-way ANOVA/LSD Test; ^2^ Kruskal–Wallis/Mann–Whitney U Test. Abbreviations: ALT, alanine aminotransferase; AST, aspartate aminotransferase; Glu, glucose; HDL-C, high-density lipoprotein cholesterol; HOMA-IR, insulin resistance test; LDL-C, low-density lipoprotein cholesterol; OB, obesity; ORL, orlistat; TBARS, thiobarbituric acid reactive substances; TC, total cholesterol; TG, triglyceride; TT, total thiol; WT, white tea.

**Table 5:** Semi-quantitative analysis result (median- 25% and 75% interquartile values).

Group	Hypertrophic Adipocytes	Infiltrative Areas	Oedematous Areas	Vascular Congestion	HPS
Control	0 (0–0)	0 (0–0)	0 (0–0)	0 (0–0)	0 (0–0)
HFD	2 (2–2) ^a^	1 (1–2) ^a^	2 (1–2) ^a^	2 (1.5–2) ^a^	2 (1.5–2) ^a^
HFD+ORL	1 (1–1.5) ^a,b^	1 (0–1) ^b^	1 (0–1) ^b,c^	0 (0–1) ^b^	0 (0–1) ^b^
HFD+WT	1 (1–1) ^a,b^	0 (0–1) ^b^	1 (1–1) ^a,d^	0 (0–1) ^b^	0 (0–1) ^b^

^a^ *p* = 0.000; compared to the control group, ^b^ *p* = 0.000; compared to the HFD group, ^c^ *p* = 0.011; compared to the control group, ^d^ *p* = 0.008; compared to the HFD group. Kruskal–Wallis/Tamhane T2 Test.

**Table 6:** Quantitative analysis results (arithmetic mean ± standard deviation).

Group	Adipocyte Superficial Area (µm^2^)
Control	384.91 ± 22.61
HFD	479.62 ± 19.12 ^a^
HFD+ORL	393.91 ± 18.81 ^b,c^
HFD+WT	401.43 ± 50.71 ^b,c^

^a^ *p* = 0.000; compared to the control group, ^b^ *p* = 0.001; compared to the control group, ^c^ *p* = 0.000; compared to the HFD group. Kruskal–Wallis/Tamhane T2 Test.

**Table 7:** Stereological analysis results (arithmetic mean ± standard deviation).

Group	Numerical Density (mm^3^)
Control	12.65 ± 2.93
HFD	32.25 ± 4.59 ^a^
HFD+ORL	24.00 ± 4.65 ^a,c^
HFD+WT	17.55 ± 2.74 ^b,c,d^

^a^ *p* = 0.000; compared to the control group, ^b^ *p* = 0.001; compared to the control group, ^c^ *p* = 0.000; compared to the HFD group, ^d^ *p* = 0.000; compared to the HFD+ORL group. One-way ANOVA/Tukey HSD Test.

**Table 8:** Semi-quantitative analysis result (median 25% and 75% interquartile values).

Group	Hypertrophic Adipocytes	Infiltrative Areas	Oedematous Areas	Vascular Congestion	HPS
Control	0 (0–0)	0 (0–0)	0 (0–0)	0 (0–0)	0 (0–0)
OB	2 (2–3) ^a^	1 (1–2) ^a^	2 (1–2) ^a^	2 (1.5–2) ^a^	2 (1.5–2) ^a^
OB+ORL	1 (0.5–1) ^a,b^	1 (0–1) ^b^	1 (0–1) ^b,c^	0 (0–1) ^b^	0 (0–1) ^b^
OB+WT50	1 (0–1) ^a,b^	0 (0–0.5) ^b^	1 (0.5–1) ^a,d^	0 (0–1) ^b^	0 (0–1) ^b^
OB+WT150	1 (1–1) ^a,b^	0 (0–1) ^b^	1 (1–1) ^a,d^	0 (0–1) ^b^	0 (0–1) ^b^

^a^ *p* = 0.000; compared to the control group, ^b^ *p* = 0.000; compared to the HFD group, ^c^ *p* = 0.011; compared to the control group, ^d^ *p* = 0.008; compared to the HFD group. Kruskal–Wallis/Tamhane T2 Test.

**Table 9:** Quantitative analysis results (arithmetic mean ± standard deviation).

Group	Adipocyte Superficial Area (µm^2^)
Control	383.81 ± 21.62
OB	477.41 ± 17.10 ^a^
OB+ORL	395.77 ± 16.54 ^a,b^
OB+WT50	402.85 ± 49.36 ^a,b^
OB+WT150	399.74 ± 49.01 ^a,b^

^a^ *p* = 0.001; compared to the control group, ^b^ *p* = 0.001; compared to the OB group. Kruskal–Wallis/Tamhane T2 Test.

**Table 10:** Stereological analysis results (arithmetic mean ± standard deviation).

Group	Adipocyte Numerical Density (×10^6^ µm^3^)
Control	1.74 ± 0.61
OB	4.17 ± 0.82 ^a^
OB+ORL	2.06 ± 0.91 ^a,b^
OB+WT50	1.76 ± 0.62 ^a,b^
OB+WT150	1.35 ± 0.54 ^a,b^

^a^ *p* = 0.001; compared to the control group, ^b^ *p* = 0.001; compared to the OB group. One-Way ANOVA/Tukey HSD Test.

## Data Availability

No datasets were generated or analyzed during the current study.
